# Development and preliminary validation of the evaluation scale for nurses’ core emergency response abilities in sudden major infectious disease outbreaks

**DOI:** 10.1186/s12912-025-02922-3

**Published:** 2025-03-10

**Authors:** Yuxin Zhu, Mengjuan Jing, Xiulu Xu, Jiamei Jing, Hanghang Jin, Jiaxin Li, Liming Li

**Affiliations:** 1 School of Nursing and Health, Henan University, Kaifeng, Henan 475004 China; 2 Department of Critical Care Medicine, Henan Provincial People’s Hospital, Zhengzhou, Henan 450000 China; 3 School of Nursing and Health, Zhengzhou University, Zhengzhou, Henan 450000 China; 4 Nursing Department, Henan Provincial People’s Hospital, Zhengzhou, Henan 450000 China; 5 Key Laboratory of Nursing and Medical Science of Henan Province, Zhengzhou, Henan 450000 China

**Keywords:** Communicable diseases, Nursing, Emergency response capabilities, Reliability, Validity

## Abstract

**Background:**

In recent years, the escalating frequency and intensity of infectious disease outbreaks have underscored the profound severity and global ramifications of public health challenges confronting healthcare systems. As frontline responders during emerging infectious disease crises, nurses’ core emergency response competencies critically influence the timeliness and efficacy of outbreak prevention and control measures. Currently, standardized assessment instruments for evaluating nurses’ core emergency response capabilities during major infectious disease outbreaks remain underdeveloped.

**Aims:**

This study aimed to construct and psychometrically validate a Core Emergency Response Competency Evaluation Scale for nurses during major infectious disease outbreaks.

**Methods:**

A convenience sample of nurses was recruited from eight tertiary hospitals in Henan Province, China. The three-phase study comprised: (1) item generation (Delphi survey and pilot testing), (2) scale development (item analysis and exploratory factor analysis), and (3) scale validation (reliability and validity assessments).

**Results:**

The finalized 45-item scale encompasses eight dimensions: Fundamental Nursing Knowledge, Legal Policy & Ethical Practice, Core Nursing Skills, Disease Assessment & Monitoring, Emergency Response Coordination, Infection Prevention & Control, Nurse Personal Attributes, and Rehabilitation & Recovery. Exploratory factor analysis demonstrated an 83.353% cumulative variance contribution for the eight-factor model. Confirmatory factor analysis indicated excellent model fit (χ²/df = 1.943, RMR = 0.020, RMSEA = 0.050, IFI = 0.951, TLI = 0.947, CFI = 0.951, PNFI = 0.835). The scale exhibited strong reliability (Cronbach’s α = 0.987, split-half = 0.956, test-retest = 0.977) and validity (I-CVI = 0.875-1.00; S-CVI = 0.974). Convergent validity was supported by AVE values (0.611–0.778) and CR values (0.903–0.955).

**Conclusion:**

The psychometrically robust Core Emergency Response Competency Evaluation Scale for Sudden Major Infectious Diseases demonstrates excellent reliability and validity, providing a scientifically validated tool for assessing nurses’ emergency preparedness in infectious disease crises.

## Background

According to the World Health Statistics Report 2022, infectious diseases remain one of the ten leading causes of death globally, posing a significant crisis to public health security [1]. Due to their unpredictability, widespread transmission, extensive harm, and life-threatening nature, infectious diseases are prone to cause large-scale infections and casualties within a short period [2]. These diseases not only inflict serious damage to public health but also impact the global economic system, threaten national security, and destabilize society through multiple pathways [3–6].

The frequency and intensity of infectious disease outbreaks in recent years underscore the profound impact of this pressing public health challenge on the global healthcare system. Since the beginning of the 21st century, major infectious diseases such as AIDS, tuberculosis, and viral hepatitis, along with various new outbreaks, have seriously threatened human life, health, and property safety [7]. Notably, the emergence of new infectious diseases, including severe acute respiratory syndrome (SARS) in 2003, influenza A (H1N1) in 2009, the Ebola virus epidemic in West Africa in 2014, novel coronavirus pneumonia in 2020, and the monkeypox epidemic in 2022, has posed significant challenges to the existing prevention and treatment systems for infectious diseases [3, 8].In this context, hospitals, as critical institutions for disease monitoring, diagnosis, and patient treatment, must urgently organize emergency response teams to execute medical rescue operations during major infectious disease outbreaks. Nursing staff, representing the largest professional group within the healthcare system, are often on the front lines of responses to new infectious disease outbreaks [9]. They assume vital functions, including pre-screening and triage, infection control, emergency rescue, and multi-sectoral collaboration. Based on experiences from emergency responses to infectious disease outbreaks, the emergency response capabilities of nursing staff are essential for effectively addressing acute and critical infectious diseases. Consequently, quantitatively assessing the core response abilities of nurses in the context of major infectious disease emergencies holds significant practical value for safeguarding patient safety, enhancing the quality of medical rescue, and optimizing public health emergency management mechanisms.

Through a systematic review of the existing literature, emergency competence evaluation tools for nurses can be classified into two main categories: (a) Disaster Preparedness and Response Questionnaires for Nurses. This category includes tools such as The Emergency Preparedness Information Questionnaire (EPIQ) [10, 11], The Disaster Preparedness Evaluation Tool (DPET) [12], and the Disaster Preparedness Questionnaire (DPQ), with the EPIQ and DPET being the most widely utilized. Prior to the global outbreak of the COVID-19 pandemic, there were relatively few studies on the emergency response capacity related to infectious disease outbreaks in foreign countries, largely due to differences in the classification of public health emergencies domestically and internationally. Consequently, the management of infectious disease outbreaks was often categorized under “disaster preparedness” and “public health emergencies.” Therefore, the aforementioned questionnaires are primarily used to assess the emergency response capacity of nurses in significant infectious disease situations. However, these assessment tools encompass a broad range of scenarios, including natural disasters, accidents, terrorist activities, and biochemical crises, as well as various dimensions such as disaster warning, injury classification, and on-site first aid. Given the specificity of infectious diseases, these questionnaires lack core indicators pertinent to the prevention and control of infectious disease outbreaks, such as biosafety protection, premonitory control, and outbreak monitoring. Thus, the applicability of these questionnaires to major infectious disease outbreaks warrants careful consideration.(b) Specific Questionnaires for Major Infectious Disease Outbreaks: In response to the cyclical epidemics of new infectious diseases, assessment tools tailored to the characteristics of specific pathogens have been developed within the academic community. For instance, during the Ebola virus disease (EVD) outbreak, researchers in South India [13], Romania [14], and Sudan [15] created questionnaires to evaluate the knowledge, attitudes, and behaviors of healthcare workers regarding EVD. Similarly, during the Middle East Respiratory Syndrome (MERS) outbreak, South Korea developed a three-dimensional scale assessing nurses’ intention, stress, and professionalism in coping with MERS [16]. Iran also constructed a tool to assess nurses’ knowledge, attitudes, and behaviors toward MERS [17]. During the H1N1 pandemic, Australia [18] and Singapore [19] released questionnaires to evaluate healthcare workers’ perceptions, attitudes, and knowledge regarding H1N1. The recent global pandemic of coronavirus-associated pneumonia (COVID-19) has also led to the development of a series of multidimensional assessment tools [20–25].

In summary, while specific questionnaires have been developed in the context of various infectious disease epidemics, most competency assessment tools are based on the contextual characteristics of the prevailing infectious disease, resulting in a relatively limited focus [26]. These tools are often not universally applicable to emerging infectious diseases. Furthermore, existing assessment tools targeting the emergency response capacity for major infectious diseases primarily address a broad range of medical personnel, whereas nurses possess significant specificity in their professional practice scope, job responsibilities, and role positioning in epidemic prevention and control. This is especially true regarding critical aspects such as patient care, emergency response, and recovery and reconstruction. Currently, no core emergency response competency assessment tools specifically designed for nurses during outbreaks of major infectious diseases have been developed, making it challenging for nursing managers to accurately assess the core emergency competencies of nurses and hindering the development of targeted training activities.

Therefore, this study aims to systematically generalize and summarize the key characteristic elements of core emergency response competence in sudden major infectious diseases through in-depth theoretical analysis and extensive literature research. Additionally, it seeks to construct a scientific and systematic evaluation scale for core emergency response competence for nurses dealing with sudden major infectious diseases using the Delphi method. This scale can serve as a self-assessment tool, enabling nurses to accurately identify their strengths and weaknesses in core emergency response competence during major infectious disease emergencies, thereby facilitating targeted improvements in their professional knowledge and skills.

Moreover, the scale will provide a reliable reference for hospitals in selecting, training, and assessing high-quality emergency nursing personnel, contributing to the continuous optimization of teaching methods and content in medical and educational institutions. Furthermore, the scale can offer valuable data support for hospitals and health administrations, assisting them in making more scientific and rational decisions regarding personnel deployment and policy formulation, particularly during infectious disease outbreaks. This will effectively aid administrators in rapidly deploying and optimizing nursing resources, enhancing the efficiency and effectiveness of emergency responses. This study focuses on scale development and reliability testing and does not involve any clinical trials. Therefore, clinical trial number: Not applicable.

## Methods

The researchers successfully obtained informed consent from the subjects with the support and assistance of the hospital administrators. The study was conducted from July 2024 to August 2024 and involved the processes of item development, scale development, and scale validation to systematically create and validate the Core Emergency Response Competency Scale for Nurses in Major Infectious Disease Emergencies. During the project development stage, we first established a preliminary item pool for the scale through literature analysis and made necessary adjustments based on expert feedback obtained during the Delphi survey. Subsequently, experts were invited to evaluate the content validity of the scale. To further assess the applicability of the items, we conducted a small pre-survey with a sample of nurses. In the scale development stage, we performed a large-scale survey of the nursing population using the initially constructed core emergency response competency evaluation scale for nurses facing major infectious disease outbreaks. The scale items were further refined through item analysis and exploratory factor analysis (EFA). In the scale validation phase, the final developed scale was tested for reliability and validity.

This study was approved by the Ethics Committee of Henan Provincial People’s Hospital (approval number: 2022-40) and strictly adhered to the relevant ethical guidelines. Consent was obtained from the heads of the hospital and departments prior to the commencement of the study. All subjects participated voluntarily with informed consent and were free to withdraw from the study at any time for any reason. We assure that participants’ personal information and data will be kept strictly confidential and used solely for research purposes.

### Phase 1 item development

#### Item generation

This study was theoretically guided by the PPRR crisis management theory [27, 28] and the ICN Disaster Nursing Core Competency Framework 2.0 [29]. The PPRR crisis management theory is a prominent framework in crisis management, indicating that the life cycle of a crisis comprises four stages: pre-crisis prevention, pre-crisis preparation, crisis outbreak response, and post-crisis recovery. The ICN Disaster Nursing Core Competency Framework 2.0 (2019) is organized into eight domains: preparedness and planning, communication, incident management systems, safety and security, assessment, intervention, recovery, and legal and ethical considerations, which is regarded as the gold standard for disaster nursing competency [30]. Based on the structure of these two theoretical frameworks, this study aimed to construct and define the dimensional set of core emergency competencies for nurses in the context of a major infectious disease outbreak.

To scientifically develop the scale item pool, the research team systematically searched and analyzed academic literature and policy guidance documents in relevant fields. Specific search strategies included terms such as “public health/ infectious diseases / communicable diseases /epidemic / COVID-19 / SARS /Ebola ”“nurse / nursing”“ability /core competence /emergency response capacity”. These terms were used to search authoritative databases, including CNKI, WanFang Data, VIP, CBM, PubMed, and Embase, to gather a comprehensive range of literature related to the prevention and control of infectious disease outbreaks among nursing staff. Additionally, we explored the official websites of the World Health Organization, the Centers for Disease Control and Prevention, and the State Council of China, along with relevant laws, regulations, policy guidelines, and emergency protocols. Through rigorous literature screening and content analysis, combined with group discussions and brainstorming sessions, we initially refined and summarized the first draft of the scale, which comprised eight dimensions and 54 items.

Subsequently, we conducted two rounds of Delphi expert correspondence to obtain evaluations of the initial entries. A total of 16 experts from the fields of clinical nursing, nursing management, and infectious disease nursing were consulted. The selection of correspondence experts is crucial for ensuring reliable consultation results [31]. In this study, the principles of representativeness, authority, and feasibility [32] were strictly adhered to when selecting experts. The inclusion criteria were as follows: (1) a bachelor’s degree or higher; (2) an intermediate title or above; (3) at least 10 years of experience in nursing management, clinical nursing, or infectious disease nursing; (4) experience in responding to infectious disease emergencies; and (5) motivation and voluntary participation in the study. A mean importance score of ≥ 4.00, a full score ratio of ≥ 0.50, and a coefficient of variation (CV) of ≤ 0.25 were established as criteria for entry screening [33]. Entries were added, deleted, and modified based on expert feedback and group discussion outcomes. Through this process, the scale was refined to include eight dimensions: Fundamental Nursing Knowledge, Legal Policy and Ethical Practice, Core Nursing Skills, Disease Assessment and Monitoring, Emergency Response, Infection Prevention and Control, Nurse Personal Attributes, and Rehabilitation and Recovery, comprising a total of 48 items.

#### Content validity test

Validity refers to the extent to which a research instrument accurately reflects the intended research concept. A total of eight experts in clinical nursing, nursing management, and infectious disease nursing were invited to evaluate the relevance of each item in the scale to its corresponding dimension, using a rating scale from 1 to 4, where 1 indicates “not relevant” and 4 indicates “very relevant.” Experts also provided suggestions for modifications to the items. The item-level content validity index (I-CVI) and the scale-level content validity index (S-CVI) were calculated based on the experts’ evaluations. Content validity is considered good when I-CVI ≥ 0.780 and S-CVI ≥ 0.900 [34].

#### Pilot survey

To ensure that the descriptions of the scale items were clear and comprehensible, this study selected 30 clinical nurses from a tertiary hospital in Zhengzhou City, Henan Province, through convenience sampling in June 2024 to conduct a pre-survey. After completing the survey, respondents provided feedback on their understanding of the scale entries. Items with unclear meanings were marked for further discussion and revision. The inclusion criteria for survey respondents were: (1) registered nurses currently practicing in clinical settings, (2) at least one year of clinical nursing experience, and (3) informed consent and voluntary participation in the study. Exclusion criteria included internship, refresher, and standardized training nurses.

### Phase 2 scale development

Nurses from 8 tertiary general hospitals in Henan Province, China, were selected as survey respondents using the convenience sampling method. The inclusion criteria for these respondents were consistent with those of the pre-survey. Considering that the sample size should be 5–10 times the number of items for factor analysis [35], a sample size of over 200 cases was required for validation factor analysis [36]. To account for a 10–20% rate of invalid questionnaires, a total of 718 nurses were recruited for this study. Among them, 30 clinical nurses from a tertiary hospital in Henan Province were conveniently selected in August 2024 to complete the questionnaire again two weeks later to assess retest reliability. The survey data from 339 clinical nurses from three tertiary hospitals in Henan Province collected in July 2024 were used for scale development, while data from 379 clinical nurses from five other tertiary hospitals in August 2024 were used for scale validation. Before the survey commenced, the research team contacted the management of the hospitals to inform them of the requirements for completing the questionnaires. The managers then notified the eligible clinical nurses to fill out the questionnaire online via Questionnaire Star. To ensure data integrity and prevent duplication, each IP address was restricted to submitting one completed questionnaire. After collection, the quality of the questionnaires was reviewed by two individuals, and invalid questionnaires were excluded. (Note: Invalid questionnaires refer to those where all items are answered with the same option, where logic is unclear, or where responses are systematically filled out.)

#### Item analysis

Item analysis was conducted to assess the discriminatory power and distinction of the scale entries. The entries were screened based on the following criteria [37], which served as a basis for potential deletion or modification: (1) Critical ratio method: Respondents’ total scores were ranked, with the top 27% classified as the high group and the next 27% as the low group. An independent samples t-test was used to compare the differences between the two groups, excluding entries with *P* > 0.05 and a critical ratio (CR) < 3.000. (2) Correlation coefficient method: Pearson correlation coefficients were calculated between the scores of each item and the total scores of the scale, excluding entries with *P* > 0.05 or absolute correlation coefficients < 0.4. (3) Cronbach’s α coefficient method: If the total Cronbach’s α coefficient of the scale increased significantly after deleting an entry, that entry was excluded.

#### Exploratory factor analysis (EFA)

Following item analysis, exploratory factor analysis was conducted using SPSS 25.0 software to determine the factor structure of the developed scale. To ensure the validity of the factor analysis, we assessed its appropriateness using Bartlett’s test of sphericity and the Kaiser-Meyer-Olkin (KMO) test. KMO coefficients greater than 0.9 indicate a very good fit; 0.8 to 0.9 indicates a good fit; 0.7 to 0.8 indicates a fair fit; 0.6 to 0.7 indicates a bare fit; and less than 0.6 indicates a poor fit for factor analysis. Bartlett’s test of sphericity was applied with *p* < 0.05. Factors were extracted using principal component analysis with maximum rotation, retaining entries with factor loadings ≥ 0.50 and no double loadings.

### Phase 3 scale validation

#### Confirmatory factor analysis (CFA)

Confirmatory factor analysis was performed using AMOS 28.0 to further evaluate the structural validity and degree of fit. Validation factor analysis utilized the maximum likelihood method to calculate the chi-square value/degrees of freedom (χ²/df), goodness-of-fit index (GFI), root mean square error of approximation (RMSEA), root mean square residual (RMR), standardized root mean square residual (SRMR), normed fit index (NFI), incremental fit index (IFI), Tucker-Lewis index (TLI), and comparative fit index (CFI) to assess model fit.

#### Convergent and discriminant validity

Composite Reliability (CR) and Average Variance Extracted (AVE) were used as indicators to evaluate convergent validity. Generally, CR > 0.7 and AVE > 0.5 indicate good convergent validity [38]. Discriminant validity was tested by comparing the square root of AVE with the correlation coefficient value; when the square root of AVE exceeds the correlation coefficient, it indicates good discriminant validity of the scale.

#### Tests of reliability

Reliability refers to the degree of consistency, accuracy, or precision of the results obtained using a specific research instrument. (1) Internal consistency reliability: Cronbach’s α coefficient and Spearman-Brown split-half reliability of the total scale and each dimension were calculated to assess scale reliability. A Cronbach’s α coefficient > 0.7 [39] and Spearman-Brown split-half reliability *r* > 0.7 indicate greater reliability. (2) External stability reliability: Retest reliability assessed the stability of the scale over time. Retest reliability was calculated for 30 participants who completed the same questionnaire within two weeks. Generally, a retest reliability ≥ 0.700 with *P* < 0.05 indicates good stability of the scale [40].

## Results

### Consultation on expert characteristics

A total of 16 experts from 13 provinces (municipalities/autonomous regions), including Beijing, Tianjin, Hebei, Liaoning, Gansu, Henan, Hubei, Hunan, Chongqing, Jiangxi, Jiangsu, Zhejiang, and the Guangxi Zhuang Autonomous Region, participated in the study. The average age of the experts was 46.94 ± 6.90 years, with an average of 26.06 ± 8.56 years of work experience. Other characteristics of the experts involved in the correspondence consultation are detailed in Table 1. The effective recovery rate of the questionnaires for both rounds of consultation was 100%. The experts’ familiarity coefficients (Cs) were 0.900 and 0.875, the judgment basis coefficients (Ca) were 0.925 and 0.938, and the authority coefficients (Cr) were 0.913 and 0.906, indicating high motivation and authority among the experts, and suggesting that the consultation results were reliable. The Kendall’s harmony coefficients for the two rounds of expert consultation were 0.282 (χ² = 238.992) and 0.297 (χ² = 223.180), respectively, both with *p* < 0.001, indicating an improvement in the degree of harmonization of experts’ opinions and supporting the credibility of the results.

**Table 1:** General information about the experts consulted

Category	Number of examples	Percentage
**Gender**		
Female	15	93.75
Male	1	6.25
**Age (years)**		
30 ~ 39	2	12.50
40 ~ 49	8	50.00
50 ~ 59	6	37.50
**Clinical career (years)**		
10 ~ 19	5	31.25
20 ~ 29	6	37.50
30 ~ 39	4	25.00
40 ~ 49	1	6.25
**Professional title**		
Senior Title	9	56.25
Vice-senior Title	6	37.50
Middle title	1	6.25
**Highest education**		
Bachelor’s degree	8	50.00
Master’s degree	6	37.50
Doctorate degree	2	12.50
**Hospital level**		
Tertiary hospitals	16	100.00

### Participant characteristics

In July 2024, the first round of the questionnaire survey yielded 402 completed questionnaires, of which 339 were valid, resulting in an effective recovery rate of 84.33%. In August 2024, the second round of the questionnaire survey yielded 436 completed questionnaires, with 379 valid questionnaires, resulting in an effective recovery rate of 86.93%. A total of 838 questionnaires were distributed across the two rounds, resulting in 718 valid questionnaires. Most participants were female (*n* = 671, 80.1%). Table 2 provides detailed characteristics of the participants.

**Table 2:** Demographic characteristics for participants

Category	Total sample (*n* = 718,%)	EFA sample (*n* = 339,%)	CFA sample (*n* = 379,%)	Test-retest sample (*n* = 30,%)
**Age (years)**				
≤ 25	40	18	22	3
26 ~ 30	134	52	82	2
31 ~ 35	260	116	144	12
36 ~ 40	178	94	84	8
≥ 41	106	59	47	5
**Gender**				
Female	671	325	346	29
Male	47	14	33	1
**Professional title**				
Nurse	50	20	30	5
Senior nurse	235	129	106	17
Supervisor nurse	394	163	231	8
Co-chief nurse	39	27	12	0
**Clinical career (years)**				
1 ~ 5	96	41	55	5
6 ~ 10	203	78	125	4
11 ~ 15	226	109	117	15
16 ~ 20	109	60	49	3
21 ~ 25	45	30	15	2
26 ~ 30	23	12	11	1
≥ 30	16	9	7	0
**Highest education**				
Junior college and below	33	13	20	28
Undergraduate course	664	314	350	2
Master’s degree or above	21	12	9	0
**Work Department**				
Internal Medicine	306	153	153	15
Surgery	135	75	60	9
Emergency	43	18	25	1
ICU	81	33	48	5
Other	153	60	93	0

### Item development

Following the first round of expert correspondence, modifications were made based on expert feedback and discussions within the working group. A new item was added: “After experiencing an outbreak of a major infectious disease, I can adapt psychologically and actively seek external help when facing difficulties.” Experts emphasized that nurses’ ability to adapt and seek help is crucial for managing the high-pressure situations generated by an outbreak, significantly protecting their work efficiency and psychological well-being during the epidemic. Five items were merged, including “I can keep myself and the outside world healthy,” as suggested by experts. Specifically, the items “I can maintain good communication with my team and have a tacit understanding of the team” and “I have the ability to communicate and coordinate with related staff and engage in inter-professional and multi-disciplinary cooperation” were combined into “I excel in communication and coordination, enabling effective inter-professional and multi-disciplinary collaboration.” Additionally, four items with a mean significance score of < 4 or a coefficient of variation (CV) > 0.25 were deleted, including “I can accurately, timely, and completely write relevant medical documents” and “I can master necessary computer operation skills and proficiently use office software for data statistics, reporting, and screening.” Furthermore, the language of eight entries was refined. In the second round of expert consultation, the average importance score of the entries ranged from 4.125 to 5.000, with CV values from 0.000 to 0.199, indicating a concentration of expert opinions. Consequently, no entries were deleted, and the language of only two entries was adjusted. After two rounds of expert correspondence, the first draft of the scale included a total of eight dimensions and 48 entries.

Subsequently, eight experts from five provinces (municipalities/autonomous regions) were invited to evaluate the relevance of the scale entries to the concepts of the corresponding dimensions, using a four-point scoring method (1: not relevant, 4: highly relevant). Based on the experts’ ratings, the item-level content validity index (I-CVI) for each item ranged from 0.875 to 1.000, and the overall content validity index (S-CVI) for the scale was 0.984, indicating that the content validity of the scale is robust.

### Findings of the pilot study

Most of the 30 nurses who completed the scale reported that the entries were clearly stated and easy to understand, with no entries added or deleted. To better align with clinical situations and enhance comprehensibility, the group provided additional interpretations for three entries based on feedback. The pre-survey results indicated that the average time taken to complete the questionnaire was 317.133 ± 109.218 s, with a minimum of 211 s and a maximum of 597 s. To ensure the quality of the study, questionnaires completed in less than 200 s were excluded based on the research group’s recommendations.

### Scale development

#### Item analysis

Item analysis was conducted on 339 valid questionnaires from the first round of the survey. The results from the critical ratio method indicated statistically significant differences between the high and low groups for each item (*P* < 0.05, t-value > 3.000). The correlation coefficient method revealed that the Pearson correlation coefficient for one item relative to the total score of the scale was 0.361 (< 0.400), suggesting poor homogeneity between the item and the overall scale, leading to its deletion following group discussion. The Cronbach’s α coefficient method showed that the total Cronbach’s α coefficient of the scale did not significantly increase after the deletion of any item. At the conclusion of the item analysis, 47 items demonstrating good discriminatory ability were retained for factor analysis.

#### Exploratory factor analysis

Exploratory factor analysis results indicated a KMO value of 0.968 and a χ² value of 21,344.364 (*P* < 0.001) for Bartlett’s test of sphericity, confirming its suitability for factor analysis. The cumulative variance contribution rate of the eight extracted factors was 82.286%, demonstrating good structural validity for the scale. The corresponding factor loading values for each entry ranged from 0.450 to 0.856, with two entries exhibiting factor loading values < 0.500, failing to meet the criteria for item attribution, and thus were deleted after group discussion. Following the removal of these two entries, exploratory factor analysis was conducted again, yielding a KMO value of 0.968, a χ² value of 20,609.846 (*P* < 0.001), and a cumulative variance contribution rate of 83.353% for the eight factors. The factor loading values for each entry under their respective factors ranged from 0.523 to 0.854, all exceeding 0.5, and no multiple loadings were observed. The final scale comprises eight dimensions and 45 entries, as detailed in Table 3.

**Table 3:** Exploratory factor analysis results (*n* = 695)

Item	Factors							
	1	2	3	4	5	6	7	8
1	0.657							
2	0.692							
3	0.647							
4	0.667							
5	0.684							
6	0.671							
7			0.854					
8			0.674					
9			0.752					
10			0.662					
11			0.600					
12			0.624					
13								0.590
14								0.558
15								0.723
16								0.754
17		0.630						
18		0.719						
19		0.659						
20		0.601						
21		0.655						
22		0.678						
23		0.529						
24						0.732		
25						0.607		
26						0.595		
27						0.610		
28						0.584		
29						0.601		
30							0.648	
31							0.670	
32							0.697	
33							0.699	
34				0.614				
35				0.716				
36				0.555				
37				0.562				
38				0.564				
39				0.727				
40					0.575			
41					0.591			
42					0.523			
43					0.567			
44					0.793			
45					0.763			

Extraction Method: Principal Component Analysis

Rotation Method: Varimax with Kaiser Normalization

a. The rotation converged after nine iterations

### Scale validation

#### Confirmatory factor analysis

**Fig. 1:**
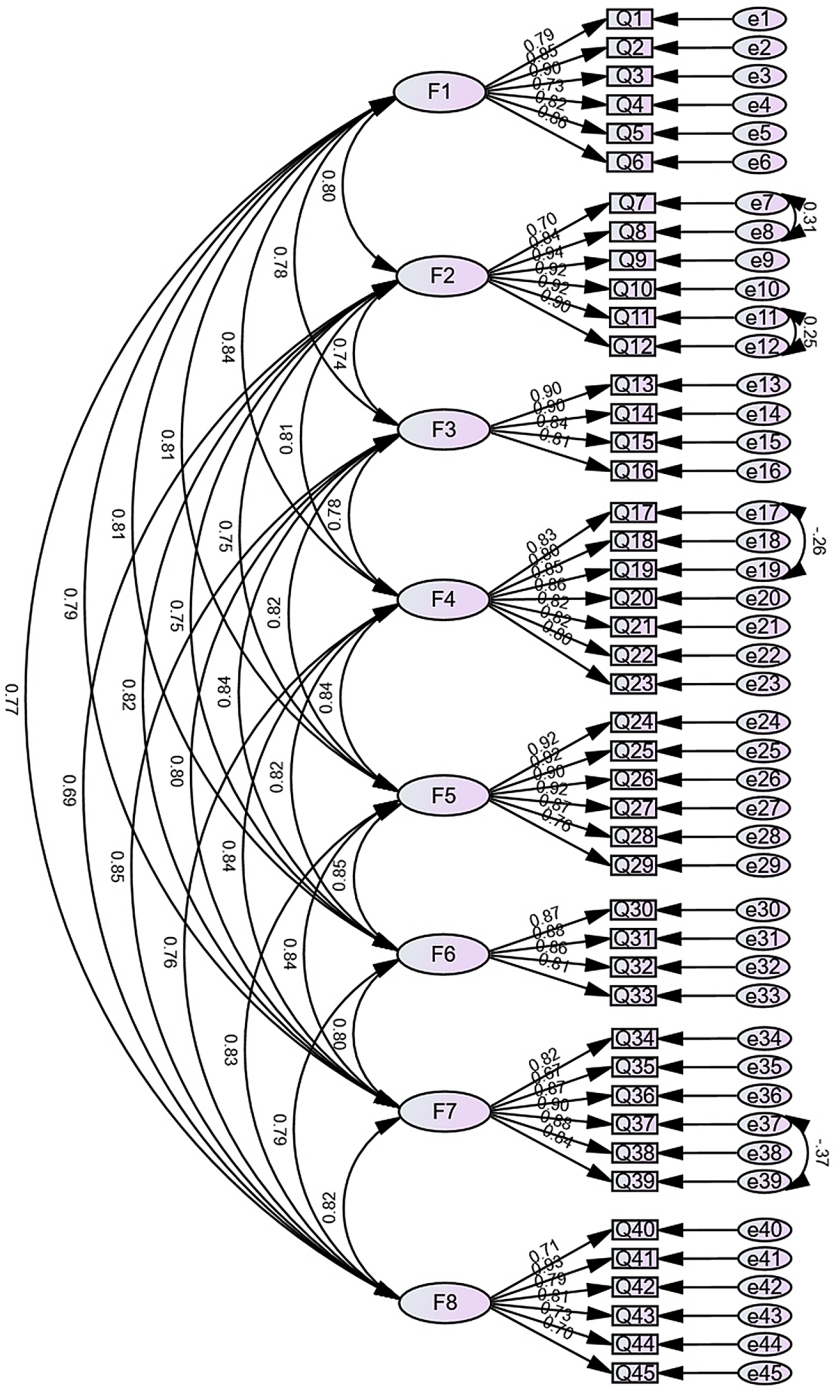
Structure equation modeling of the scale. Note: F1 = Fundamental Nursing Knowledge, F2 = Legal, Policy, and Ethical Practice, F3 = Core Nursing Skills, F4 = Disease Assessment and Monitoring, F5 = Emergency response to emergencies, F6 = Infection Prevention and Control, F7 = Nurse Personal Attributes, F8 = Rehabilitation and Recovery, Q1-45 = Item1-45 (see Table 7)

CFA was employed to validate the factor structure derived from EFA. Preliminary results from the validation model revealed relatively large modification indices (MIs) between e7 and e8, e11 and e12, e17 and e19, and e37 and e39. Significant covariance was noted among these items, indicating shared variance in measuring highly similar aspects of the target constructs. To address this empirical evidence of shared method variance, we adhered to psychometric conventions by establishing correlated residuals in the CFA models [41]. Consequently, covariance was introduced between the aforementioned entries to refine the models. The adjusted individual model fit indices were χ²/df = 1.943 < 3.000, RMSEA = 0.050 < 0.080, CFI = 0.951 > 0.900, NFI = 0.905 > 0.900, GFI = 0.828 > 0.800, RMR = 0.828 > 0.900, SRMR = 0.0336 < 0.050, IFI = 0.951 > 0.900, TLI = 0.947 > 0.900, and PNFI = 0.835 > 0.500 (see Table 4). The standardized factor loading model diagram generated from the validation factor analysis is illustrated in Fig. 1, with all entries exhibiting factor loadings greater than 0.40 and statistical significance (*P* < 0.05), indicating good structural validity. The correlations between the dimensions are presented in Table 5. The path coefficients of the initial hypothesis model are shown in Table 6. The final scale developed in this study is displayed in Table 7.

**Table 4:** Results of the confirmatory factor analysis model fit indices (*n* = 379)

Parameters	Indicator level
χ²/df	1.943 (<3.000)
RMSEA	0.050 (<0.080)
CFI	0.951 (>0.900)
NFI	0.905 (>0.900)
GFI	0.828 (>0.808)
RMR	0.020 (<0.050)
SRMR	0.0336 (<0.050)
IFI	0.951 (>0.900)
TLI	0.947 (>0.900)
PNFI	0.835 (>0.500)

**Table 5:** The correlation between domains and the total score

Domains	1	2	3	4	5	6	7	8	Total
1	1								
2	0.745	1							
3	0.697	0.611	1						
4	0.787	0.731	0.750	1					
5	0.738	0.690	0.759	0.816	1				
6	0.747	0.638	0.758	0.740	0.822	1			
7	0.779	0.738	0.720	0.843	0.805	0.731	1		
8	0.695	0.656	0.796	0.793	0.812	0.763	0.740	1	
Total	0.880	0826	0.852	0.925	0.912	0.866	0.903	0.888	1

Note: 1 =(Fundamental Nursing Knowledge), 2 = (Legal, Policy, and Ethical Practice), 3 = (Core Nursing Skills), 4 = (Disease Assessment and Monitoring), 5 = (Emergency response to emergencies), 6 = (Infection Prevention and Control), 7 = (Nurse Personal Attributes), 8 = (Rehabilitation and Recovery)

**Table 6:** Path coefficients of the initial hypothesis model

Name			Estimate	SE	CR	*P* value
F7	<-->	F8	0.292	0.030	9.592	***
F6	<-->	F8	0.278	0.029	9.545	***
F5	<-->	F8	0.310	0.031	10.008	***
F4	<-->	F8	0.245	0.026	9.249	***
F3	<-->	F8	0.307	0.031	10.018	***
F2	<-->	F8	0.224	0.027	8.419	***
F1	<-->	F8	0.267	0.029	9.151	***
F6	<-->	F7	0.344	0.033	10.396	***
F5	<-->	F7	0.382	0.035	10.960	***
F4	<-->	F7	0.332	0.032	10.474	***
F3	<-->	F7	0.352	0.033	10.578	***
F2	<-->	F7	0.323	0.034	9.639	***
F1	<-->	F7	0.335	0.034	9.969	***
F5	<-->	F6	0.385	0.034	11.271	***
F4	<-->	F6	0.321	0.031	10.521	***
F3	<-->	F6	0.363	0.033	10.989	***
F2	<-->	F6	0.291	0.031	9.361	***
F1	<-->	F6	0.343	0.033	10.266	***
F4	<-->	F5	0.350	0.032	10.990	***
F3	<-->	F5	0.379	0.034	11.247	***
F2	<-->	F5	0.310	0.032	9.620	***
F1	<-->	F5	0.363	0.034	10.562	***
F3	<-->	F4	0.309	0.030	10.366	***
F2	<-->	F4	0.288	0.030	9.549	***
F1	<-->	F4	0.325	0.032	10.256	***
F2	<-->	F3	0.293	0.031	9.428	***
F1	<-->	F3	0.336	0.033	10.217	***
F1	<-->	F2	0.306	0.033	9.343	***
e7	<-->	e8	0.077	0.014	5.332	***
e11	<-->	e12	0.021	0.006	3.450	***
e17	<-->	e19	− 0.041	0.010	-4.253	***
e37	<-->	e39	− 0.044	0.008	-5.654	***

Note: ***P < 0.001

**Table 7:** The final perceived social support scale

Domain/ item number	Item text
**A Fundamental Nursing Knowledge**	
1	I am familiar with the definitions, types, epidemiological characteristics, transmission routes, and prevention measures for common major infectious diseases
2	I understand the treatment principles and nursing considerations for common major infectious diseases
3	I can safely administer medications to patients according to the doctor’s instructions
4	I know how to correctly use protective equipment for various infectious diseases
5	I am aware of the types and procedures for vaccinations against common infectious diseases
6	I understand coping strategies for sudden infectious disease outbreaks in specific situations.(e.g.: response to sudden infectious diseases after bioterrorist attacks/natural disasters)
**B Legal**,** Policy**,** and Ethical Practice**	
7	I am informed about the latest laws, regulations, and policies related to major infectious disease emergencies
8	I am familiar with the statutory reporting timelines and processes for various infectious disease emergencies
9	I adhere to ethical principles when addressing major national infectious disease emergencies
10	I can quickly adapt to temporary medical teams and their working environments
11	I can promptly respond to the decisions and requirements of government and organizations
12	I am knowledgeable about the emergency plan for major infectious disease outbreaks
**C Core Nursing Skills**	
13	I can maintain proficiency in various nursing practices even in constrained rescue environments or while wearing personal protective equipment
14	I can collect, store, and safely transport specimens according to relevant infectious disease technical guidelines
15	I can standardize the use of common instruments and equipment and promptly address instrument alarms(e.g.: Simple respirators, ECG monitors, defibrillators, micropumps, ventilators, etc.)
16	I can implement critical care rescue techniques(e.g.: irway management, and assistance with mechanical ventilation, prone ventilation, ECMO, etc.)
**D Disease Assessment and Monitoring**	
17	I can quickly identify issues in the work environment or workflow and implement countermeasures to mitigate risks
18	I can efficiently extract key nursing information from a patient’s infectious disease history, physical examination, laboratory results, and other relevant materials
19	I can anticipate potential complications and nursing risks in patients with infectious diseases through thorough nursing assessments
20	I can dynamically assess the effectiveness of nursing care based on changes in the condition of patients with infectious diseases and adjust nursing strategies promptly
21	I can make predictive nursing decisions informed by my professional knowledge and past experiences
22	I can identify trends in abnormal health status among individuals or groups by recognizing typical features and clinical manifestations of infectious diseases
23	During infectious disease response efforts, I can monitor my health status and proactively report any abnormalities
**E Emergency response to emergencies**	
24	I can prioritize care and allocate medical resources effectively
25	I can accurately execute the response procedures for infectious disease incidents
26	I can quickly triage and manage an influx of infectious disease patients, ensuring safe transport
27	I can swiftly identify and appropriately resettle at-risk vulnerable groups(e.g.: the elderly, children, pregnant women, and individuals with disabilities)
28	I can effectively address a range of sudden and complex issues(e.g.: such as occupational exposure, accidental spillage of specimens)
29	In emergencies, I can conduct rescue operations effectively(e.g.: in cases of cardiac arrest, asphyxia, air embolism, or drug allergies)
**F Infection Prevention and Control**	
30	I am knowledgeable about the principles and methods for isolation, quarantine, and disinfection of major infectious diseases
31	I understand that the “three zones and two channels” zoning principle ensures the orderly diversion of healthcare providers, patients, and logistics
32	I can effectively implement infection prevention and control measures(e.g.: managing medical waste, decontaminating bodies of patients with confirmed or suspected infectious diseases)
33	I can effectively implement standard precautions and hierarchical protection(e.g.: hand hygiene, proper use of protective equipment, and safe injection practices)
**G Nurse Personal Attributes**	
34	I maintain good physical and mental fitness, enabling me to endure high-intensity nursing work
35	I possess a strong sense of responsibility and mission, and I am willing to actively participate in frontline care
36	I excel in communication and coordination, enabling effective collaboration across disciplines
37	I can utilize communication skills to engage with individuals from diverse cultural backgrounds and age groups, including non-verbal communication
38	I can identify gaps in my knowledge or skills and actively seek to learn about the latest information and technologies related to sudden infectious diseases
39	I actively participate in training and emergency drills related to various infectious diseases
**H Rehabilitation and Recovery**	
40	I can provide emergency health education and rehabilitation guidance to the public regarding various infectious diseases
41	I can assess and address the physical and mental health needs of individuals and families at different stages, facilitating referrals or follow-up as necessary
42	After a major infectious disease emergency, I can adjust my mindset and seek external help when needed
43	I can promptly summarize and share experiences from practice or drills related to infectious disease prevention and control to guide other personnel
44	I can contribute to the development and updating of nursing practice guidelines and emergency plans for major infectious diseases
45	I employ evidence-based scientific thinking and possess a sense of innovation, enabling me to participate in relevant scientific research

#### Convergent and discriminant validity

The average variance extracted (AVE) values ranged from 0.611 to 0.778, all exceeding 0.5, while the composite reliability (CR) values ranged from 0.903 to 0.955, all surpassing 0.7, indicating strong convergent validity. The majority of dimensions met the standards for discriminant validity; however, the square root of AVE for some dimensions was slightly lower, resulting in acceptable discriminant validity [42] (see Table 8).

**Table 8:** Result of discriminant validity

Construct	1	2	3	4	5	6	7	8
1	**0.829**							
2	0.796	**0.875**						
3	0.783	0.739	**0.862**					
4	0.843	0.807	0.776	**0.824**				
5	0.812	0.749	0.821	0.844	**0.882**			
6	0.814	0.747	0.836	0.822	0.850	**0.857**		
7	0.791	0.825	0.804	0.845	0.837	0.801	**0.835**	
8	0.766	0.694	0.853	0.757	0.826	0.787	0.822	**0.782**

Note: The bold numbers along the diagonal represent the square root of the Average Variance Extracted (AVE)

#### Tests of reliability

The Cronbach’s α coefficients for each dimension of the scale developed in this study ranged from 0.928 to 0.975, with a total Cronbach’s α coefficient of 0.987, indicating that the scale meets the criteria for an ideal measurement tool [43]. The split-half reliability values for the dimensions ranged from 0.915 to 0.960, with a total split-half reliability of 0.956. Additionally, 30 survey participants were randomly selected for retesting, yielding retest reliabilities for the dimensions ranging from 0.788 to 0.924, and a total scale retest reliability of 0.977. This indicates that the scale demonstrates good internal consistency and external stability [44]. The Pearson correlation coefficients between the scale and its dimensions are shown in Table 9.

**Table 9:** Reliability analysis results (*n* = 339)

Dimension	Reliability coefficient		
	Cronbach’s α coefficient	Split-half reliability	Test–retest reliability
Total scale	0.987	0.956	0.977**
1	0.937	0.924	0.796**
2	0.930	0.915	0.793**
3	0.928	0.926	0.855**
4	0.975	0.960	0.856**
5	0.962	0.960	0.813**
6	0.949	0.959	0.788**
7	0.948	0.946	0.920**
8	0.941	0.917	0.924**

Note: **P < 0.01;1 =(Fundamental Nursing Knowledge), 2 = (Legal, Policy, and Ethical Practice), 3 = (Core Nursing Skills), 4 = (Disease Assessment and Monitoring), 5 = (Emergency response to emergencies), 6 = (Infection Prevention and Control), 7 = (Nurse Personal Attributes), 8 = (Rehabilitation and Recovery)

## Discussion

This study developed an instrument to evaluate nurses’ core emergency competencies in the context of major infectious disease outbreaks, resulting in a scale comprising eight dimensions with 45 entries. The eight dimensions include: Fundamental Nursing Knowledge, Legal Policy and Ethical Practice, Core Nursing Skills, Disease Assessment and Monitoring, Emergency Response Skills, Infection Prevention and Control, Nurse Personal Attributes, and Rehabilitation and Recovery.

In this study, 45 items were developed through item development, scale development, and scale validation. The initial draft of the scale, containing 48 entries, was created through literature analysis, Delphi expert correspondence, and a pilot survey.

During the scale development process, three items were eliminated based on item analysis and EFA exclusion criteria. The EFA results indicated that the Nurse Competency Scale (NPCS) included eight dimensions: Fundamental Nursing Knowledge, Legal Policy and Ethical Practice, Core Nursing Skills, Disease Assessment and Monitoring, Emergency Response Skills, Infection Prevention and Control, Nurse Personal Attributes, and Rehabilitation and Recovery, explaining 83.353% of the total variance.

In the scale validation phase, preliminary results of the validation model revealed relatively large modification indices (MIs) among several entries. Based on these modification indices, six covariance correlations were added, with each correlation existing between the residuals of different items within the same dimension, with no cross-dimensionality observed. This aligns with the pre-specified model, and these correlations can be reasonably justified. After correction, the individual model fit indices were χ²/df = 1.943 < 3.000, RMSEA = 0.050 < 0.080, CFI = 0.951 > 0.900, NFI = 0.905 > 0.900, GFI = 0.828 > 0.800, RMR = 0.828 > 0.900, SRMR = 0.0336 < 0.050, IFI = 0.951 > 0.900, TLI = 0.947 > 0.900, and PNFI = 0.835 > 0.500, all within acceptable ranges. The content validity index (I-CVI) for the 45 entries in the scale ranged from 0.875 to 1.000, with an overall content validity index (S-CVI) of 0.984, meeting the requirements for content validity and indicating that the scale possesses good content validity.

The results of this study suggest that the scale developed is a valid and reliable assessment tool for measuring nurses’ core emergency response competencies during major infectious disease outbreaks. The overall process of scale development adhered to established procedures, and the methodology employed was rigorous and scientific, effectively ensuring the reliability of the scale.

## Limitations and perspectives

This study has several limitations. First, during the Delphi expert correspondence process, although the majority of expert opinions were considered for revisions, the process remained somewhat subjective, and individual suggestions from certain experts may have been overlooked [45]. Second, the nurses recruited for this study were solely from 8 hospitals in Henan Province, limiting the representativeness of the sample and potentially introducing bias into the study results. Finally, the initial scale construction relied primarily on local databases, which may introduce cultural or contextual biases that could affect the scale’s applicability and accuracy across different cultural contexts.

To address these limitations, future research should expand the scope of the study by conducting multi-center, large-sample surveys to comprehensively validate the applicability of the scale across various regions and cultural contexts. This approach will further enhance the reliability and validity of the scale and provide a more accurate foundation for evaluating nurses’ core emergency competencies on a global scale.

## Conclusions

In the public health context of frequent outbreaks of major infectious diseases, systematic assessment of nurses’ core emergency response competencies has become essential for improving the level of medical emergency preparedness. The core emergency response competency assessment tool developed in this study for nurses during major infectious disease outbreaks encompasses the entire cycle of “prevention-response-recovery.” It includes eight core dimensions: basic nursing knowledge of infectious diseases, legal policy and ethical practice, core nursing skills, assessment and monitoring abilities, emergency response capabilities, infection prevention and control skills, personal characteristics of nurses, and recovery and reconstruction abilities. This tool can comprehensively assess the core emergency response competencies of clinical nurses facing outbreaks of major infectious diseases. It not only provides a scientific basis for medical institutions to objectively identify areas for improvement in nurses’ emergency response capabilities across the hospital but also encourages nurses to enhance their professionalism through a dynamic assessment mechanism. This has a direct impact on optimizing the training pathways for infectious disease nursing personnel and constructing a highly resilient emergency response nursing team. The widespread adoption and application of this scale will contribute to strengthening nursing responses to public health emergencies through capacity building.

## Data Availability

The data that support the findings of this study are available from the corresponding author.
